# Frontotemporal dementia caused by CHMP2B mutation is characterised by neuronal lysosomal storage pathology

**DOI:** 10.1007/s00401-015-1475-3

**Published:** 2015-09-10

**Authors:** Emma L. Clayton, Sarah Mizielinska, James R. Edgar, Troels Tolstrup Nielsen, Sarah Marshall, Frances E. Norona, Miranda Robbins, Hana Damirji, Ida E. Holm, Peter Johannsen, Jørgen E. Nielsen, Emmanuel A. Asante, John Collinge, The FReJA consortium, Adrian M. Isaacs

**Affiliations:** Department of Neurodegenerative Disease, UCL Institute of Neurology, Queen Square, London, WC1N 3BG UK; MRC Prion Unit, UCL Institute of Neurology, Queen Square, London, WC1N 3BG UK; Cambridge Institute for Medical Research, University of Cambridge, Cambridge, CB2 0XY UK; Neurogenetics Research Laboratory, Department of Neurology, Danish Dementia Research Centre, Rigshospitalet, University of Copenhagen, Copenhagen, Denmark; Laboratory for Experimental Neuropathology, Department of Pathology, Randers Hospital, 8930 Randers NØ, Denmark; Institute of Clinical Medicine, Aarhus University, 8000 Aarhus C, Denmark; Memory Clinic, Department of Neurology, Danish Dementia Research Centre, Rigshospitalet, University of Copenhagen, Copenhagen, Denmark; Department of Cellular and Molecular Medicine, Section of Neurogenetics, The Panum Institute, University of Copenhagen, Copenhagen, Denmark

**Keywords:** CHMP2B, FTD, ESCRT, Lysosome, Lysosomal storage disorder

## Abstract

Mutations in the charged multivesicular body protein 2B (*CHMP2B*) cause frontotemporal dementia (FTD). We report that mice which express FTD-causative mutant CHMP2B at physiological levels develop a novel lysosomal storage pathology characterised by large neuronal autofluorescent aggregates. The aggregates are an early and progressive pathology that occur at 3 months of age and increase in both size and number over time. These autofluorescent aggregates are not observed in mice expressing wild-type *CHMP2B*, or in non-transgenic controls, indicating that they are a specific pathology caused by mutant *CHMP2B*. Ultrastructural analysis and immuno- gold labelling confirmed that they are derived from the endolysosomal system. Consistent with these findings, *CHMP2B* mutation patient brains contain morphologically similar autofluorescent aggregates. These aggregates occur significantly more frequently in human *CHMP2B* mutation brain than in neurodegenerative disease or age-matched control brains. These data suggest that lysosomal storage pathology is the major neuronal pathology in FTD caused by *CHMP2B* mutation. Recent evidence suggests that two other genes associated with FTD, *GRN* and *TMEM106B* are important for lysosomal function. Our identification of lysosomal storage pathology in FTD caused by CHMP2B mutation now provides evidence that endolysosomal dysfunction is a major degenerative pathway in FTD.

**Electronic supplementary material:**

The online version of this article (doi:10.1007/s00401-015-1475-3) contains supplementary material, which is available to authorized users.

## Introduction

Frontotemporal dementia (FTD) is the second most common form of young-onset dementia [17, 32]. FTD is characterised by atrophy of the frontal and temporal lobes, which results in alterations in personality, behaviour or language [26, 28]. FTD also shows clinical overlap with amyotrophic lateral sclerosis (ALS) and FTD and ALS share common pathologies and genetic causes [3].

Mutations in a number of genes are known to cause familial FTD. Mutations in the genes that encode tau (*MAPT*), progranulin (*GRN*) and *C9orf72* are the most common causes of FTD, whilst rare mutations have been identified in valosin-containing protein (*VCP*), TDP-43 (*TARDBP*), fused in sarcoma (*FUS*) and charged multivesicular body protein 2B (*CHMP2B*) [33]. It is currently unclear how defects in such diverse genes result in specific degeneration of neurons of the frontal and temporal lobes.

A mutation in *CHMP2B* is responsible for an autosomal dominant form of inherited FTD (termed FTD-3) in a Danish cohort [24, 35]. The mutation occurs in a splice acceptor site, resulting in the production of two variants of C-terminally truncated CHMP2B proteins: CHMP2B^Intron5^ in which the final 36 amino acids are replaced with a single valine residue, or CHMP2B^∆10^ in which the final 36 amino acids are replaced with 29 nonsense residues [35]. A subsequent study in a Belgian FTD cohort identified a familial FTD patient with a distinct truncation mutation CHMP2B^Q165X^ that leads to the loss of the final 49 amino acids, providing further evidence that C-terminal truncations of CHMP2B lead to FTD [20, 41].

CHMP2B functions as a subunit of the endosomal sorting complex required for transport III (ESCRT-III). ESCRTs are multi-subunit complexes, highly conserved from yeast to mammals, which mediate the bending and fission of membranes [18]. Membrane manipulation by ESCRT complexes occurs through the sequential recruitment of the multimeric complexes ESCRTS 0 through III, which function to scaffold membrane deformation, and finally recruit VPS4 to fission the deformed membranes. This process of membrane bending is used for several biologically diverse but topologically similar processes: during the final stages of cell division, during virus egress from cells, and importantly for this study, during the maturation of endosomes [18]. Maturing endosomes acquire numerous intraluminal vesicles (ILVs) during their progress to late endosomes, which are also known as multivesicular bodies (MVBs). MVBs ultimately fuse with lysosomes to allow degradation of the endosomal content [18, 27]. In addition, MVBs fuse with autophagosomes to form hybrid compartments termed amphisomes, which then fuse with lysosomes, enabling degradation [5, 27]. Mutant CHMP2B^Intron5^ has been shown to affect the maturation of both endosomes and autophagosomes [10, 22, 25, 38], implicating impaired lysosomal degradation as a key pathway in FTD caused by CHMP2B mutation. Importantly, accumulating evidence suggests that progranulin, a common genetic cause of FTD, and TMEM106B a major risk factor for FTD also have roles in endolysosomal function [7, 9, 16, 21, 34, 36, 37], with progranulin mutations leading to pathology reminiscent of lysosomal storage disorders [16].

We have previously shown that transgenic mice expressing endogenous levels of human C-terminally truncated mutant CHMP2B^Intron5^ show progressive gliosis and p62 inclusion pathology, which are also observed in *CHMP2B* mutation patient brain [14, 19]. Here we report, using immunoblotting and light and electron microscopy, that these CHMP2B^Intron5^ mice additionally develop a separate and distinct progressive lysosomal storage pathology that is characterised by large autofluorescent aggregates. These autofluorescent aggregates are not seen in CHMP2B^wild-type^ transgenics, or non-transgenic control mice. Importantly, we also report increased autofluorescent aggregates in the frontal cortex of FTD-3 patients with the CHMP2B mutation, when compared to neurodegenerative disease controls. These data indicate that impaired lysosomal storage is a novel pathological pathway in the aetiology of FTD caused by CHMP2B mutation, and provide evidence that lysosomal degradation is a key pathway in FTD pathogenesis.

## Materials and methods

### Mice

The mutant CHMP2B^Intron5^ expressing mouse line Tg153 was used and maintained as a homozygous line as previously described [14]. The full-length human CHMP2B^Wild-type^ mouse line Tg168 was maintained as a hemizygous line and non-transgenic littermates used for controls as previously described [14].

### Mouse brain immunofluorescence and pathology quantification

**Fig. 1:**
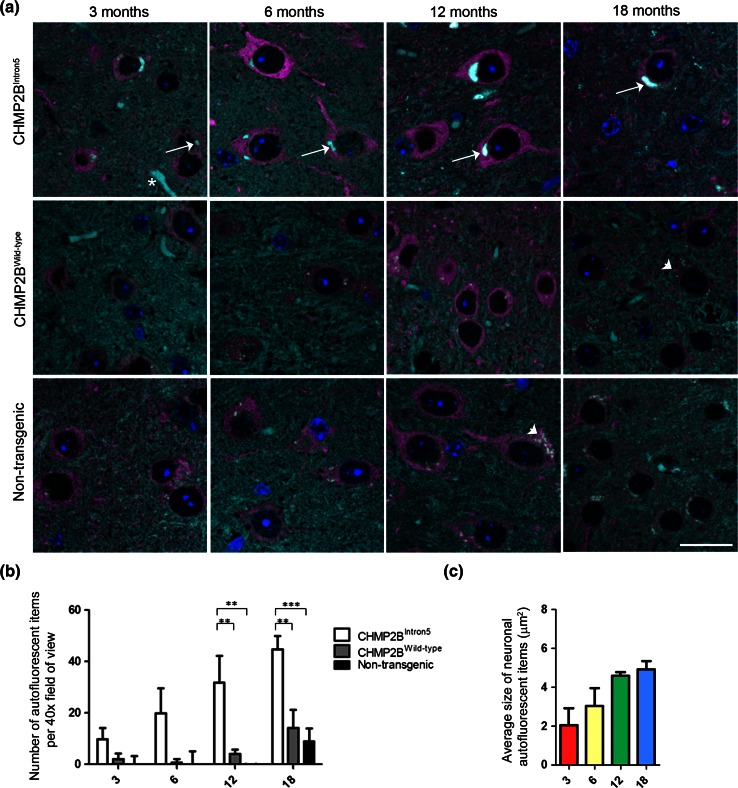
Progressive accumulation of autofluorescent aggregates in CHMP2B^Intron5^ mice. **a** Spectrally unmixed images showing autofluorescent aggregates in the thalamus of CHMP2B^Intron5^ mice, but not in CHMP2B^Wild-type^ or non-transgenic control samples at 3, 6, 12 or 18 months of age. Nuclei are stained with DAPI (*blue*), neurons with β-III tubulin (*magenta*) and autofluorescence is shown in *cyan*. *Arrows* indicate autofluorescent aggregates, *arrowheads* indicate lipofuscin and an *asterisk* indicates a blood vessel. *Scale bar* 20 μm. **b** Quantification of the number of autofluorescent items in the thalamus of CHMP2B^Intron5^, CHMP2B^Wild-type^ and non-transgenic control mice. **c** Average size of neuronal autofluorescent items at 3, 6, 12 and 18 months in the thalamus of CHMP2B^Intron5^ mice. Data are shown as mean ± SEM. Significance was determined using a two-way ANOVA, with post hoc Bonferroni test. *p < 0.05, **p < 0.01, ***p < 0.001. All statistically significant differences are shown

Mouse brains were immersion fixed in 10 % buffered formal saline, embedded in paraffin wax and sections cut at 3–4 μm thickness. Antigen retrieval was performed by microwaving for 20 min in 0.1 M citrate buffer. Primary antibodies were as follows: p62 (GP62-C, PROGEN), β-III tubulin (ab78078, Abcam), Iba1 (019-19741, Wako), Iba1 (ab5076, Abcam), GFAP (Z0334, DAKO), SMI-94 (Ab24567, Abcam) and CAII (ab6621, Abcam). Alexa Fluor-conjugated secondary antibodies (Life Technologies) were used to visualise the staining. Images were collected using a 40× lens with 1.4 NA on a Zeiss LSM 710, or a 63× lens with a 1.4 NA on a Zeiss LSM 510 confocal microscope. Spectral imaging with subsequent linear unmixing was used to separate the autofluorescence from the emission of each of the Alexa Fluor-labelled secondary antibodies. Quantification was performed blinded on three mice per genotype (CHMP2B^Intron5^, CHMP2B^Wild-type^ and non-transgenics) at 3, 6, 12 and 18 months of age. Six 40× images were taken from the thalamus and cortex of each sample. Volocity software (Perkin-Elmer) was used to automatically identify fluorescent populations, with images individually thresholded to remove background noise. Blood vessels were a specific background autofluorescence that were also identified by this image analysis protocol (for example see asterisk in Fig. 1a). To control for this interference, the background number of autofluorescent items was set as the average number of autofluorescent items in non-transgenic mice at 3 months of age, and was subtracted from all autofluorescent item counts. Cellular localisation of autofluorescent items was determined manually based on their presence within either Iba1- or β-III tubulin-positive cells. Statistical analysis was performed with Graphpad Prism 5 software, using either a two-way or a one-way ANOVA with post hoc Bonferroni tests analysing selected pairs of columns, or a Student’s *t* test, as indicated in the figure legends.

### Cases

Brain specimens (described in Table 1) were obtained from the Queen Square Brain Bank for Neurological Disorders, UCL Institute of Neurology; the MRC London Neurodegenerative Diseases Brain Bank, Institute of Psychiatry, King’s College London; the Division of Neuropathology, UCL Institute of Neurology, and the Frontotemporal Dementia Research in Jutland Association. Paraffin-embedded frontal cortex sections (7 μm) were analysed for FTD-3 (*n* = 5), Alzheimer’s disease pathology (*n* = 3) and age-matched neurologically normal controls (*n* = 5). The study was approved by the UCL Institute of Neurology and National Hospital for Neurology and Neurosurgery Local Research Ethics Committee.

**Table 1:** Details of cases used in this study

Diagnosis	Sex	Age at death	Postmortem interval
Control	M	71	5
Control	M	61	53
Control	F	68	9
Control	M	57	79
Control	F	68	45
AD	M	79	96
AD	F	65	30
AD	F	79	38
FTD-3	F	61	Unknown
FTD-3	F	76	Unknown
FTD-3	M	54	30
FTD-3	F	87	18
FTD-3	F	69	18

### Human brain immunofluorescence and pathology quantification

Imaging was performed using standard confocal multi-channel imaging, with autofluorescence identified by its presence in all channels. Immunofluorescence staining and autofluorescent object identification were performed as above for mouse sections. Quantification of pathology was performed on all cases by a researcher blinded to the sample origin. Autofluorescent objects were manually categorised based on their characteristic morphology (see Figs. 7, S8), enabling differentiation of granular lipofuscin from the large, dense autofluorescent aggregates. Autofluorescent objects were localised to either microglia (identified by Iba1 positive staining), or neurons (identified by their characteristic large nuclei).

### Conventional electron microscopy

Mice aged to either 6 or 18 months were perfusion fixed with 4 % paraformaldehyde (PFA) and the brains removed and post-fixed overnight in the same fixative. 100 μm thick sections were cut using a vibratome. Areas of interest were dissected and refixed with 2 % PFA/2.5 % glutaraldehyde in 0.1 M cacodylate buffer. The tissue was then post-fixed with 1 % osmium tetroxide before being dehydrated with ethanol, and embedded in Araldite epoxy resin (Agar scientific, UK).

Ultrathin sections (70 nm) were cut using a diamond knife mounted to a Reichert Ultracut S ultramicrotome (Leica, UK) and picked up onto coated EM grids. The sections were stained with lead citrate and observed in a FEI Tecnai Spirit (Eindhoven, Netherlands) transmission electron microscope at an operating voltage of 80 kV. In conventional electron microscopy, neurons were identified by their characteristic large nuclei with a clear nucleolus and electron lucent cytoplasm, as observed in NeuN immunogold-labelled cells. Oligodendrocytes were identified by their characteristic darker cytoplasm.

### Pre-embedding labelling of mouse brains for electron microscopy

Mouse brains were fixed and dissected as described above. Thick sections were washed with HEPES buffered saline (HBS) pH 7.4 before being permeabilised in HBS containing 0.05 % Triton x-100 and 10 % BSA. Tissue was incubated with primary antibodies overnight at 4 °C in HBS with 0.005 % Triton x-100 and 1 % BSA. Primary antibodies used were as follows: LAMP1 (1D4B, University of Iowa Hybridoma Bank), LAMP2 (Abl-93, University of Iowa Hybridoma Bank), p62 (GP62-C, PROGEN), β-III tubulin (D71G9, cell signalling), NeuN (ABN78, Millipore), GFAP (Z0334, Dako) and Iba1 (ab5076, Abcam). Tissue was then washed before being incubated with either anti-mouse or anti-rabbit nanogold secondaries (Nanoprobes, USA). The tissue was washed before being refixed with 2 % PFA/2.5 % glutaraldehyde in 0.1 M cacodylate buffer. Nanogold particles were then enhanced using Goldenhance (Nanoprobes, USA). Tissue was then post-fixed with 1 % osmium tetroxide and processed for resin embedding as described above.

### Immunoblotting

The olfactory bulb and cerebellum were removed from whole brains, and homogenates prepared from the remainder in Dulbecco’s phosphate-buffered saline containing complete EDTA-free protease inhibitors (Roche) using a TissueRuptor (Qiagen) to make a 10 % w/v solution. Following a 2-min 200×*g* spin to pellet debris, the supernatant was combined 1:1 with 2 % sarkosyl (*N*-lauroylsarcosine) in D-PBS. Benzonase (Novagen) was added to digest DNA and the homogenates were incubated with constant agitation at 37 °C for 1 h. 2× Laemmli sample buffer was added and the samples were heated at 100 °C for 10 min prior to sodium dodecyl sulphate polyacrylamide gel electrophoresis. Samples were run on 4–12 % or 10 % Bis’-Tris gels (Life Technologies) with MOPS buffer, then transferred onto polyvinylidene fluoride, blocked with 5 % bovine serum albumin in PBS-T, and probed using either anti-LAMP-1 (1D4B, University of Iowa) or anti-cathepsin-D (sc-6486, Santa Cruz Biotechnology), respectively. Loading controls were performed using mouse anti-β-actin (A5441, Sigma). Detection was performed with horseradish peroxidase-conjugated secondary antibodies and SuperSignal West Pico Chemiluminescent Substrate (Thermo Scientific). Quantification was performed using ImageJ software. Band intensity was normalised to the indicated loading control, and averages taken of three mice per genotype.

## Results

### Autofluorescent aggregates are a specific and progressive pathology in CHMP2B^Intron5^ mice

We observed that CHMP2B^Intron5^ mice developed autofluorescent aggregates that fluoresce at all spectra of excitation/emission. Lambda imaging and spectral unmixing were used to identify all autofluorescent objects in brain sections from CHMP2B^Intron5^, CHMP2B^wild-type^, or non-transgenic mice, in combination with β-III tubulin staining to identify neurons at 3, 6, 12 or 18 months of age. We focused on the thalamus (Fig. 1a) and cortex (Fig. S1) as these are the main sites of pathology in CHMP2B^Intron5^ mice [14]. We noted two morphologically distinct types of autofluorescent material in neurons: (i) age-related lipofuscin depositions, which are present in all mice and have a granular dot-like appearance (arrowheads in Fig. 1a); and (ii) large, dense aggregates which are only present in CHMP2B^Intron5^ mice (arrows in Fig. 1a). We next performed automated analysis of the number of autofluorescent items. Our automated analysis removed the smallest autofluorescent items using a size cutoff but was not able to effectively distinguish between the large dense autofluorescent aggregates and lipofuscin, due to the clumping of the lipofuscin dots. Therefore, lipofuscin deposits were also counted and as expected accumulated in non-transgenic and CHMP2B^wild-type^ mice by 18 months of age (Fig. 1b). Due to the additional presence of the large autofluorescent aggregates, CHMP2B^Intron5^ mice had higher numbers of autofluorescent items at all ages (Fig. 1b). This was confirmed by plotting the size distribution of all detected autofluorescent items, which showed that as CHMP2B^Intron5^ mice increase in age, they had a significantly greater proportion of larger autofluorescent items than non-transgenic and CHMP2B^wild-type^ mice, even with the limitation that large lipofuscin clumps common to all genotypes were also counted (Fig. S2). Further quantification showed that the CHMP2B^Intron5^ autofluorescent aggregates increase in size and frequency with age (Fig. 1b, c).

### Autofluorescent aggregates are distinct from p62 inclusions in CHMP2B^Intron5^ mice

**Fig. 2:**
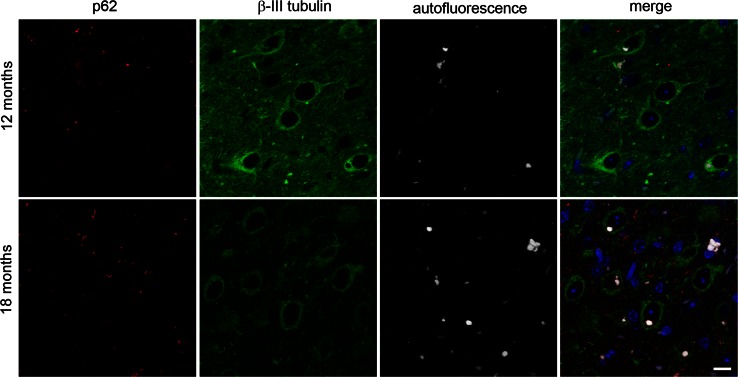
Autofluorescent aggregates and p62-positive inclusions are distinct pathologies. Spectrally unmixed images of p62 staining (*red*) and autofluorescence (*white*) in the thalamus of 12- and 18-month-old CHMP2B^Intron5^ mice shows little overlap between p62 and autofluorescence. Neurons are stained with β-III tubulin (*green*), and nuclei with DAPI (*blue*). *Scale bar* 10 μm

To assess the relationship between this autofluorescent pathology and the p62 inclusions we have previously reported in CHMP2B^Intron5^ mice [14], we analysed their co-occurrence in the thalamus (Fig. 2) and the cortex (Fig. S3). Both types of pathology were found to occur frequently in the cortex and thalamus of CHMP2B^Intron5^ mice, but without obvious overlap in localisation. This clearly indicates that the p62 inclusions and autofluorescent aggregates are distinct pathologies. However, in addition to the discrete p62 inclusions, occasionally autofluorescent aggregates could be detected that were surrounded by a halo of p62 (for example see Fig. S3, arrow in upper inset), which may indicate an attempt to target the aggregates for degradation.

### CHMP2B^Intron5^ autofluorescent aggregates occur in neurons and microglia

**Fig. 3:**
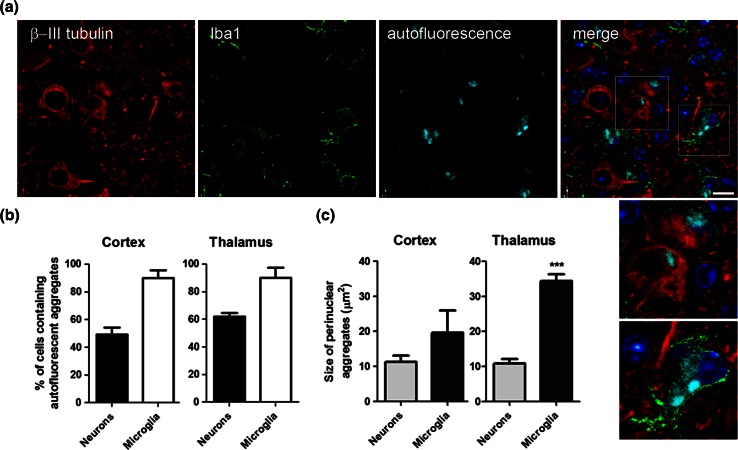
Autofluorescent aggregates are found in neurons and microglia in CHMP2B^Intron5^ mice. **a** Cell-type specific staining in the thalamus of 18-month-old CHMP2B^Intron5^ brain shows that autofluorescent aggregates (*cyan*) can be localised to neurons (β-III tubulin—*red*) or microglia (Iba1—*green*). Nuclei are stained with DAPI (*blue*). The *dotted boxes* in the merged image are enlarged in the *panel below*. *Scale bar* 10 μm. **b** Quantification of the percentage of neurons or microglia containing autofluorescent aggregates in the cortex and the thalamus of 18-month CHMP2B^Intron5^ mice. **c** Quantification of the size of perinuclear autofluorescent aggregates in neurons or microglia from the cortex and the thalamus of 18-month CHMP2B^Intron5^ mice. Data are shown as mean ± SEM. Significance was determined using a Student’s *t* test. ***p < 0.001. All statistically significant differences are shown

We next determined the cellular localisation of CHMP2B^Intron5^ autofluorescent aggregates using cell-type specific markers. Autofluorescent aggregates were found to predominantly localise to either neurons or microglia (Fig. 3a) but not to astrocytes (Fig. S4). This is in contrast to the localisation of p62 inclusions, which we found to be predominantly localised to oligodendrocytic processes (Fig. S5), but not neurons, astrocytes or microglia (Figs. 2, S4, S6). Oligodendrocyte localisation was consistent with the prominent association of p62 inclusions with axonal tracts, such as those in the white matter of the corpus callosum (Fig. S7). The presence of p62 inclusions and autofluorescent aggregates in different cell types further confirmed that they are distinct pathologies. By 18 months, approximately half of the neurons in the cortex contained autofluorescent aggregates (49.3 ± 5.8 %, Fig. 3b). In the thalamus, an even greater proportion of neurons contained autofluorescent aggregates (61.9 ± 4.9 %). Microglia also had a very high burden of autofluorescent pathology: 89.8 ± 6.9 % in the cortex, and 89.8 ± 9.3 % in the thalamus, at 18 months of age. In the thalamus, at 18 months, the autofluorescent microglial aggregates were significantly larger than the neuronal aggregates, by approximately threefold (Fig. 3c). A similar but non-significant trend towards larger microglial aggregates was also observed in the 18-month cortex. These data show that autofluorescent aggregates are found extensively in microglia and neurons and are the major neuronal pathology in CHMP2B^Intron5^ mice.

### CHMP2B^Intron5^ neuronal aggregates are membrane-bound and associated with the endolysosomal system

**Fig. 4:**
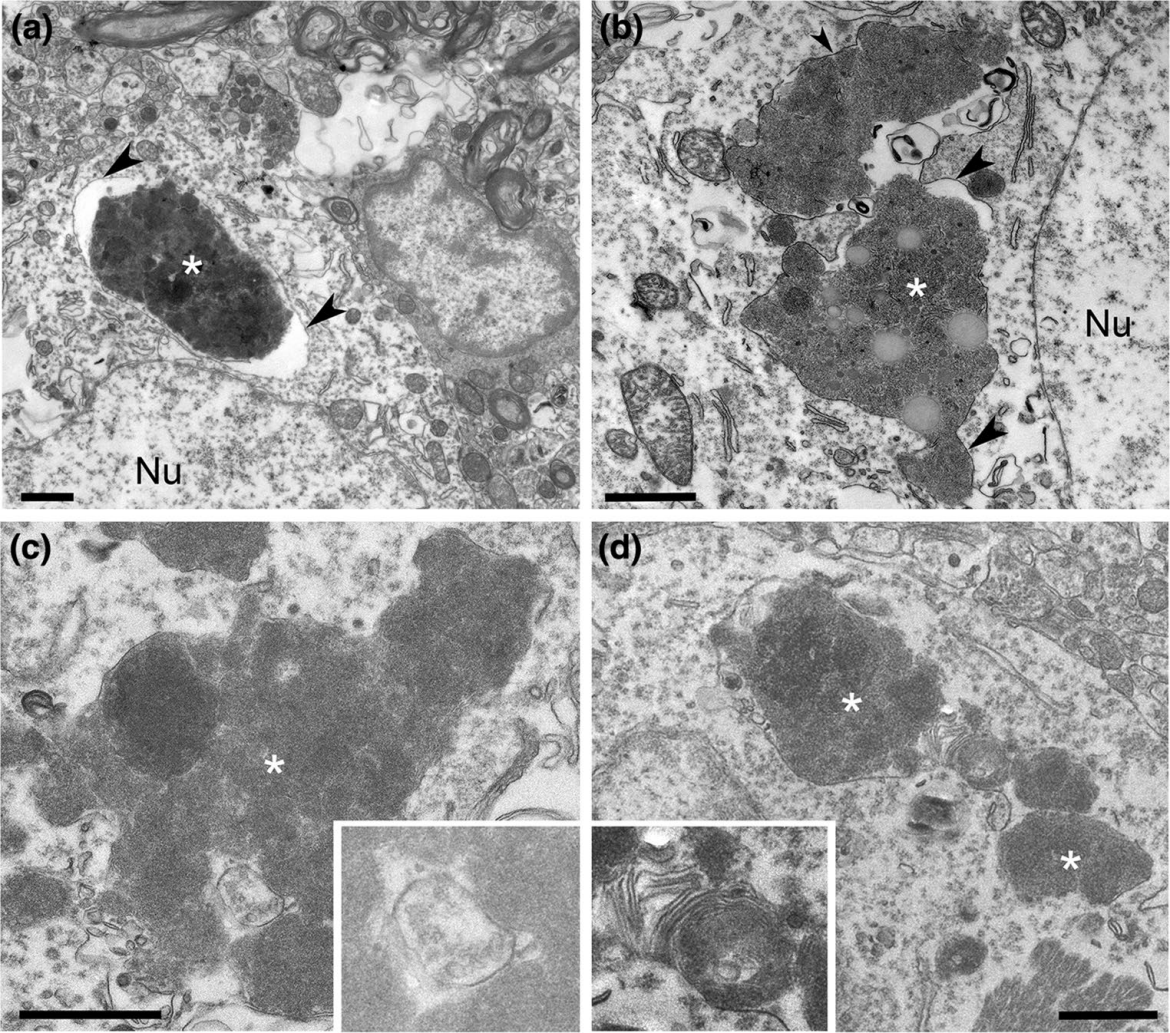
CHMP2B^Intron5^ mouse brains display large intraneuronal deposits. 18-month CHMP2B^Intron5^ mouse brains were prepared for conventional EM. Four examples of large deposits are shown (*asterisks* **a**–**d**), located next to large neuronal nuclei (Nu). These deposits were often single membrane-bound (*arrowheads*) and occasionally contained features suggestive of endocytic origin, such as multivesicular bodies (**c** *inset*) or lamellar membrane whorls typical of late endosome/lysosomes (**d** *inset*). *Scale bars* 1 μm

**Fig. 5:**
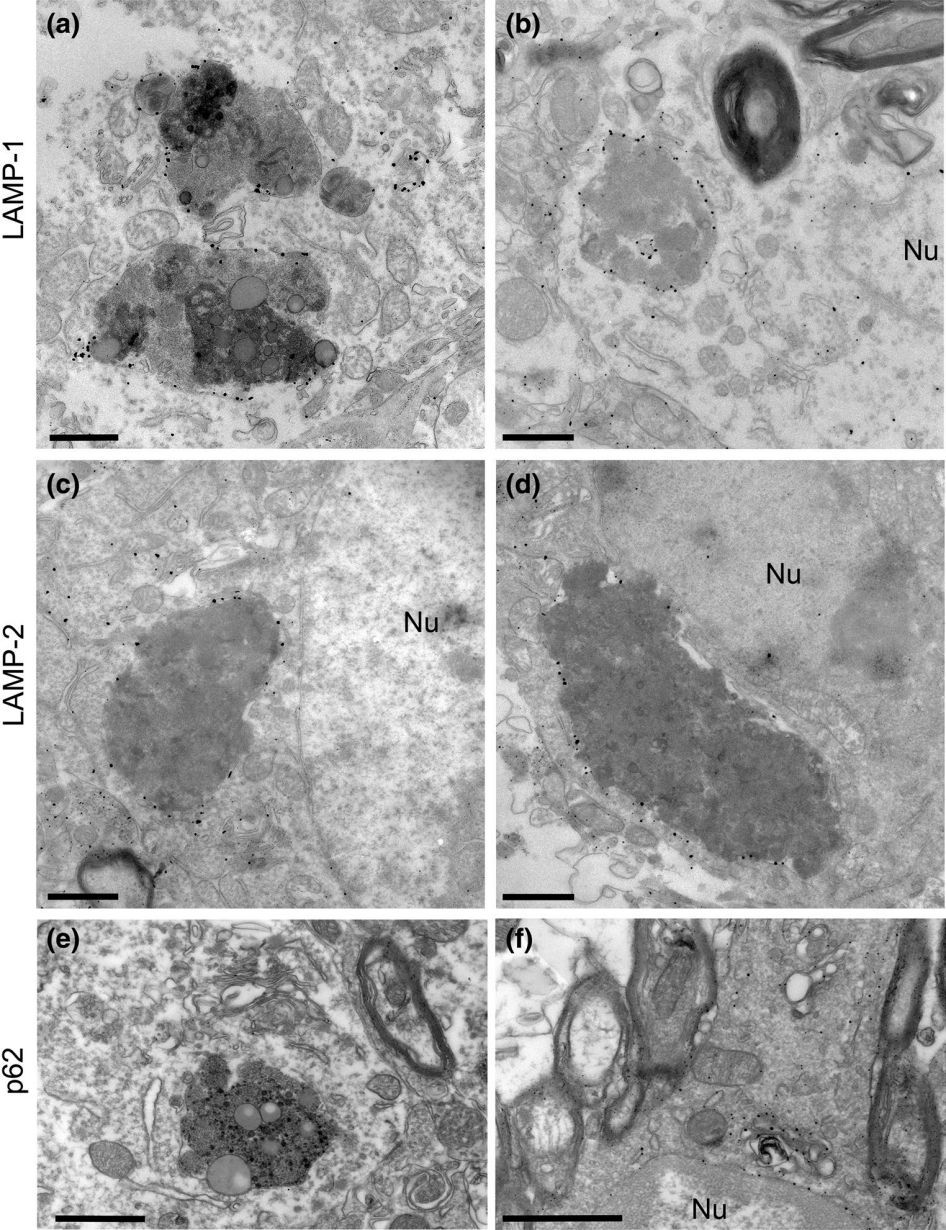
Intraneuronal deposits are of endolysosomal origin. 18-month CHMP2B^Intron5^ mouse brains were prepared for pre-embedding immuno-EM. Neuronal deposits stained positively for LAMP-1 (**a**, **b**) or LAMP-2 (**c**, **d**), but not for p62 **(e)**, although p62-positive structures could be observed in oligodendrocytes **(f)**. *Nu* nucleus. *Scale bars* 1 μm

To determine the ultrastructure of deposits in CHMP2B^Intron5^ mice, conventional electron microscopy (EM) was performed on the cortex and thalamus of 18-month-old CHMP2B^Intron5^ and non-transgenic mice. Consistent with our light microscopy data, CHMP2B^Intron5^ mice displayed numerous large aggregates within neurons (asterisks, Fig. 4), whilst smaller and less frequent lipofuscin-like deposits were observed in non-transgenic mice (average areas: CHMP2B^Intron5^ 3.99 ± 3.33 μm^2^, *n* = 38 deposits; non-transgenic 0.77 ± 0.42 μm^2^, *n* = 35 deposits, mean ± SD). CHMP2B^Intron5^ aggregates occasionally contained lipid droplets (Fig. 4b). Interestingly, the CHMP2B^Intron5^ aggregates were single membrane-bound (arrowheads Fig. 4a, b) and were often in close association with late endosomal/lysosomal structures such as multivesicular bodies (inset in Fig. 4c) and compartments containing characteristic membrane whorls (inset in Fig. 4d). To determine the precise nature of the membrane surrounding aggregates in CHMP2B^Intron5^ mouse brains, we performed pre-embedding labelling electron microscopy. Conventional EM analysis suggested that aggregates were of an endocytic origin, so the late endosome/lysosome markers LAMP-1 and LAMP-2 were tested. Both epitopes were found to be present on the membranes surrounding neuronal aggregates (Fig. 5a–d). Membrane-bound compartments containing aggregated material were much larger than normal LAMP1/2 organelles. The neuronal aggregates were negative for p62 labelling (Fig. 5e), although some p62 membrane staining could be observed on small aggregates in oligodendrocytes (Fig. 5f), consistent with our light microscopy data (Figs. 2, S5). The ultrastructural examination of the large neuronal inclusions indicates that they are derived from the deposition and build-up of lysosomal components, as is characteristically seen in lysosomal storage disorders which typically present with an accumulation of autofluorescent material [31].

### Accumulation of lysosomal proteins in the brains of CHMP2B^Intron5^ mice

**Fig. 6:**
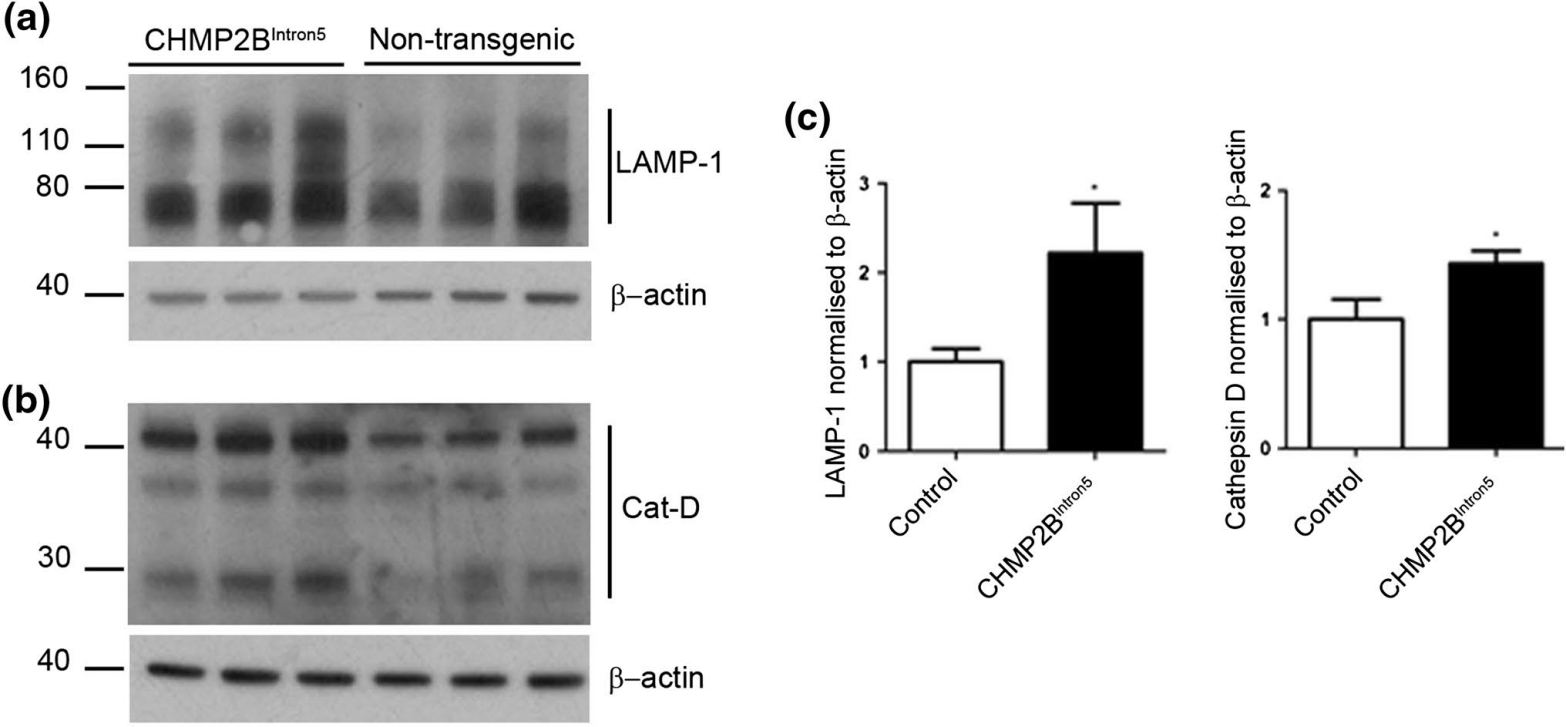
Accumulation of lysosomal proteins in CHMP2B^Intron5^ mouse brain. 18-month-old CHMP2B^Intron5^ mouse brain homogenates were immunoblotted for components of the lysosome known to accumulate in lysosomal storage disorders. **a** LAMP-1 and **b** cathepsin D were both found to be elevated in aged CHMP2B^Intron5^ mouse brains compared to non-transgenic controls **(c)**. Data are shown as mean ± SEM. Significance was determined using a Student’s *t* test. *p < 0.05. Actin blots confirm equal loading

Our data are suggestive of a lysosomal storage-type pathology characterised by autofluorescent aggregates in CHMP2B^Intron5^ mice, so we next investigated whether components of the lysosome were accumulating in brains from these mice. A significant accumulation of the lysosomal proteins LAMP-1 and cathepsin D was observed at 18 months of age in the brains of CHMP2B^Intron5^ mice compared to non-transgenic controls (Fig. 6). This indicates that FTD caused by CHMP2B mutation recapitulates aspects of lysosomal storage disorder pathology.

### Autofluorescent aggregates accumulate abnormally and selectively in the cortex of FTD-3 patients

**Fig. 7:**
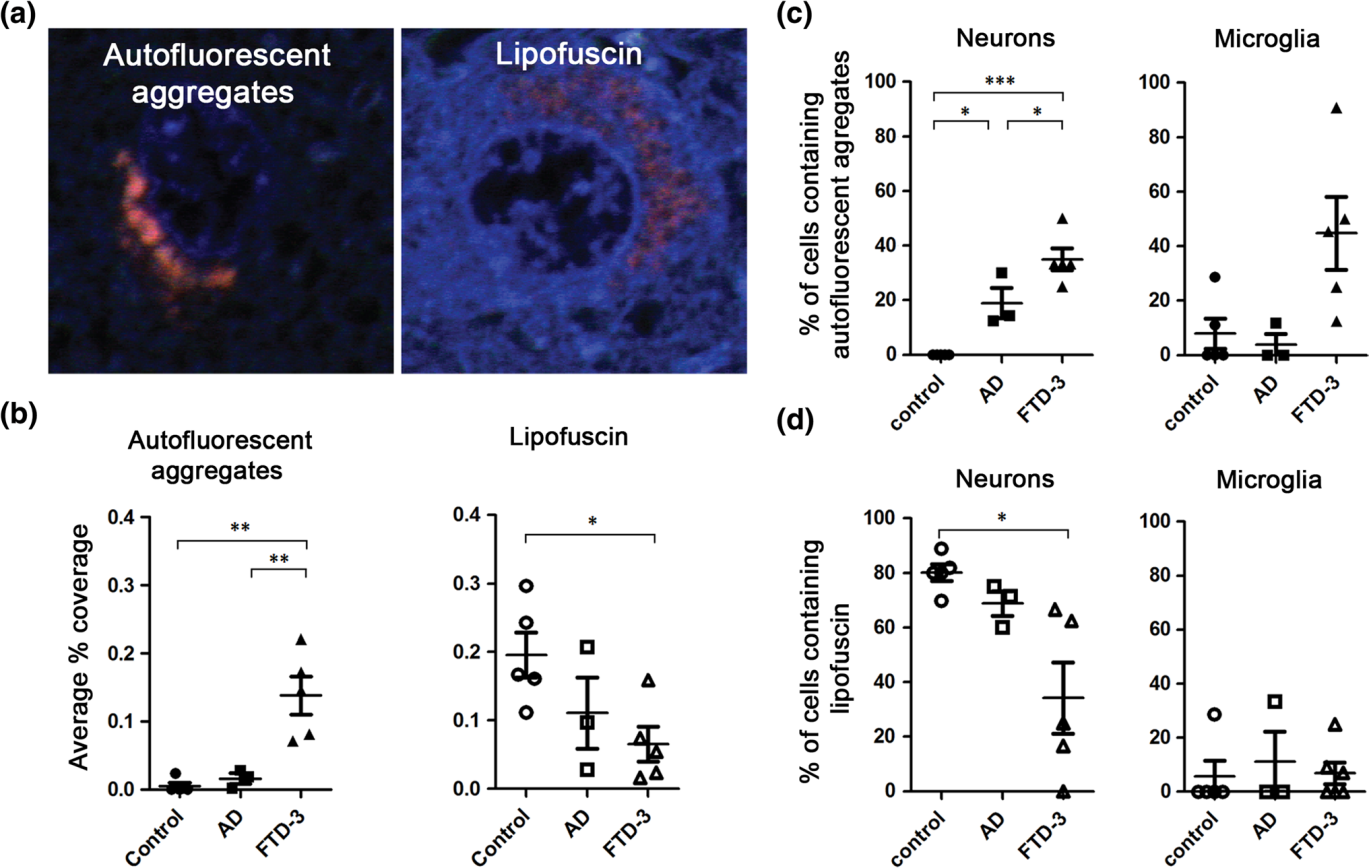
Autofluorescent aggregates are a characteristic pathology of FTD-3 patient brain. Autofluorescent pathology in frontal cortex tissue from FTD-3, AD and neurologically normal control brains was investigated. **a** Examples of an autofluorescent aggregate in FTD-3 patient frontal cortex, and the morphologically distinct granular lipofuscin in control cortex. DAPI labels the nuclei (*blue*). **b** Blinded quantification of the coverage of autofluorescent aggregates or lipofuscin per field of view in control, AD or FTD-3 patient frontal cortex. Percentage of either neurons or microglia containing autofluorescence **(c)** or lipofuscin **(d)**. Data are shown as *individual symbols per case with bars* representing mean ± SEM. *Closed symbols* autofluorescent aggregates, *open symbols* lipofuscin. Significance was determined using a one-way ANOVA, with post hoc Bonferroni test. *p < 0.05, **p < 0.01, ***p < 0.001. All statistically significant differences are shown

To assess the relevance of the autofluorescent aggregates found in CHMP2B^Intron5^ mice to human FTD-3 with CHMP2B mutation, we investigated the occurrence of autofluorescence in the frontal cortex of FTD-3 patient tissue. To assess specificity, we also examined tissue from brains with Alzheimer’s disease pathology, as a neurodegenerative disease control, and neurologically normal age-matched control samples (Table 1). As our analysis software was unable to distinguish between lipofuscin and the autofluorescent aggregates, a researcher blinded to sample origin separated each autofluorescent item identified by the software into distinct categories based on their distinctive morphology: large, dense autofluorescent aggregates or the more granular lipofuscin (Figs. 7a, S8). The tissue coverage of autofluorescent aggregates in frontal cortex was highest in FTD-3 and significantly greater than both neurologically normal and Alzheimer’s disease (AD) controls (Fig. 7b). Cell-type specificity was also analysed in the patient tissue. The percentage of neurons containing autofluorescent aggregates was also significantly higher in FTD-3 cases versus controls (Fig. 7c). Extensive lipofuscin was prevalent in all samples, as expected of aged brain tissue (Fig. 7b, d). Autofluorescent aggregates were not detected in neurologically normal control neurons, suggesting that our morphological analysis was able to distinguish this novel autofluorescent pathology from age-related lipofuscin autofluorescence. Although no significant difference was seen in the total coverage of autofluorescent aggregates between AD and controls (Fig. 7b), the percentage of neurons containing autofluorescent aggregates in AD cases was significantly greater (Fig. 7c), indicating that AD neurons contain small autofluorescent aggregates. This is consistent with suggestions that endolysosomal dysfunction occurs in the pathogenesis of AD [30]. We further analysed the hippocampus which does not show extensive degeneration in FTD-3, and did not observe large autofluorescent aggregates (Fig. S8). The occurrence of autofluorescent aggregates in FTD-3 CHMP2B mutation frontal cortex, combined with their early and progressive accumulation in CHMP2B^Intron5^ mice indicates that the build-up of lysosomally derived material is a characteristic pathology of this disease.

## Discussion

We have shown that in addition to p62 inclusions, the CHMP2B^Intron5^ mouse model of FTD exhibits a progressive autofluorescent aggregate pathology that is distinct from normal lipofuscin autofluorescence associated with ageing. These autofluorescent aggregates occur in neurons of the cortex and thalamus from 3 months of age. Aggregates increase in size and number over time, and by 18 months approximately half of neurons contain an autofluorescent aggregate. Our data clearly demonstrate that these autofluorescent aggregates are the major neuronal pathology in CHMP2B^Intron5^ mice, as the p62-positive inclusions are not generally observed in neurons. Whilst little colocalisation was seen between the previously described p62 inclusions and the autofluorescent aggregates, occasionally a halo of p62 staining could be seen surrounding the autofluorescent aggregates. This may indicate p62 deposition on the limiting membrane of the inclusion, signalling an attempt by the cell to label the inclusion for degradation. Similarly, the marked gliosis which we have previously reported in these mice [14] may indicate an attempt by microglia to clear neurons which have acquired a large burden of autofluorescent pathology. In agreement with this possibility, nearly all microglia in 18-month-old CHMP2B^Intron5^ mice contain autofluorescent aggregates, which are significantly larger in size than those found in neurons.

Ultrastructural analysis of the autofluorescent aggregates indicates that they are endosomal in origin, as they contain elements of multivesicular bodies and membrane whorls characteristic of late endosomal–lysosomal compartments. Immunoblotting also confirmed the accumulation of endolysosomal proteins in 18-month-old CHMP2B^Intron5^ mice. FTD-causing C-terminal truncation mutants of CHMP2B have been modelled in several systems, and have been found to cause perturbations in the endolysosomal and autophagy systems. Overexpression of CHMP2B^Intron5^, CHMP2B^Δ10^ or CHMP2B^Q165X^ in human neuroblastoma cells results in a characteristic enlarged endosomal phenotype [38, 41], which is also observed in both FTD-3 and CHMP2B^Q165X^ patient fibroblasts [29, 38] and FTD-patient brain [38]. Overexpression of CHMP2B^Intron5^ in HeLa cells or primary cortical neurons leads to an accumulation of autophagosomes [10, 22], which may be due to ESCRT-III having a role in the closure of autophagosome membranes [25]. However, we did not observe an obvious increase in double membrane autophagosome structures by electron microscopy in CHMP2B^Intron5^ mouse brain, arguing against this being the primary effect of CHMP2B^Intron5^. In a separate study, neither enlarged endosomes nor autophagosome accumulation were observed when CHMP2B^Intron5^ or CHMP2B^Δ10^ was expressed in primary hippocampal neurons [2]. We have now analysed the effect of expression of a physiologically relevant level of mutant CHMP2B on the neuronal endolysosomal system in vivo, and show that this results in an accumulation of autofluorescent endolysosomally derived deposits.

Crucially, similar autofluorescent aggregates can also be detected in the brain of patients with FTD-3, indicating that accumulation of endolysosomally derived intermediates is a hallmark of FTD caused by mutation in CHMP2B. FTD-3 patients were found to have a significantly greater coverage of autofluorescent aggregates compared to age-matched and neurodegenerative disease controls. The autofluorescent aggregates in both CHMP2B^Intron5^ mice and FTD-3 patient brain were morphologically distinct from lipofuscin associated with normal ageing, which is also autofluorescent and lysosomally derived. Interestingly, a concomitant decrease was seen in the lipofuscin coverage in FTD-3 patients. This could be due to an exacerbation of the natural process of age-related lipofuscinosis leading to the much larger and denser deposits that we identify as autofluorescent aggregates. Another possibility is that CHMP2B^Intron5^-induced autofluorescent aggregate formation and lipofuscin formation are distinct processes that compete for lysosomal components. We have previously reported the presence of p62-positive inclusions in FTD-3 patient brain, which predominantly occur in the hippocampus and are rare in the frontal cortex [19]. In contrast, the autofluorescent pathology was present in the frontal cortex and rare/absent in the hippocampus, indicating the two pathologies rarely co-occur. As FTD-3 brains exhibit gross atrophy of the frontal cortex with no neurodegeneration in the hippocampus [19], the autofluorescent lysosomal storage pathology, rather than the p62 inclusions, correlates best with neurodegeneration in FTD-3.

This is not the first evidence of a lysosomal pathology in FTD. Studies on mutations in *GRN*, the gene encoding progranulin, have already suggested a phenotypic overlap between FTD and lysosomal storage disorders. A pair of siblings suffering from neuronal ceroid lipofuscinosis (NCL) was found to have a homozygous mutation in *GRN* [36], and the same *GRN* mutation in the heterozygous state is known to cause FTD [4]. Close examination of *Grn*-deficient mice has revealed that these mice recapitulate features of both FTD and NCL: aged *Grn* knockout mice display an accumulation of proteins which are characteristically seen in NCL [16], and also display increased lipofuscin [1]. However, as lipofuscin and the autofluorescent aggregates we observe in CHMP2B^Intron5^ mice are morphologically distinct, it is not clear whether a common upstream pathogenic pathway is shared or whether distinct neurodegenerative pathways converge on the lysosome. In addition to these mouse studies, FTD–GRN patients were also found to have elevated levels of characteristic NCL storage components [16]. Interestingly, TMEM106B, a well-replicated risk factor for FTD [11, 12, 39, 40, 42], is localised to the lysosome [7, 9, 21], and has been implicated in the neuronal trafficking of endolysosomal structures [34, 37]. Our finding that FTD caused by CHMP2B mutation is characterised by neuronal lysosomal storage pathology now provides evidence that endolysosomal dysfunction is major pathway in the aetiology of FTD.

Whilst the accumulating evidence that lysosomal dysfunction is a major pathway in FTD indicates that it is likely to be an important neurodegenerative pathway mediated by CHMP2B^Intron5^, it does not rule out a role for other mechanisms, such as an effect on dendritic spine maintenance and function, which have also been reported [2, 6, 8]. In addition, multivesicular bodies, late endosomes and specifically ESCRTs have also been shown to play a role in the life cycle of microRNAs [15, 23], and it was recently reported that microRNA-124 is decreased in another CHMP2B^Intron5^ mouse model, leading to an alteration in AMPA receptor composition, which may also contribute to aspects of the disease phenotype [13].

We have shown here that mutant CHMP2B causes the pathological accumulation of endolysosomal components early in the disease course. Why defects in the endolysosomal system specifically lead to degeneration of the subset of neurons that are lost in FTD-3 is unclear. Cortical neurons may be particularly sensitive to perturbations in the endolysosomal equilibrium, such as alterations in endolysosomal trafficking in axons and dendrites. The *CHMP2B* mutation will be a powerful tool for investigating perturbations in neuronal endolysosomal dynamics and may provide insight into the earliest events occurring in the development of FTD.

## Electronic supplementary material

Supplementary material 1 (PDF 1531 kb)
