# Supplementary material for: Frontotemporal dementia caused by CHMP2B mutation is characterised by neuronal lysosomal storage pathology
Source: Acta Neuropathol. 2015 Sep 10;130(4):511–23. doi: 10.1007/s00401-015-1475-3 (PMC4575387; doi:10.1007/s00401-015-1475-3)
Supplement: Supplementary file 1 — Supplementary material 1 (PDF 1531 kb) [file 401_2015_1475_MOESM1_ESM.pdf]

Frontotemporal dementia caused by CHMP2B mutation is characterised by neuronal lysosomal storage pathology

Acta Neuropathologica

Emma L Clayton[*], Sarah Mizielinska[*], James R Edgar, Troels Tolstrup Nielsen , Sarah Marshall, Frances E Norona, Miranda Robbins, Hana Damirji, Ida Holm, Peter Johannsen, Jørgen E Nielsen, Emmanuel A Asante, John Collinge, the FReJA consortium and Adrian M Isaacs.

Corresponding author:

Dr Adrian Isaacs

Department of Neurodegenerative Disease, UCL Institute of Neurology, Queen Square, London WC1N 3BG

a.isaacs@prion.ucl.ac.uk

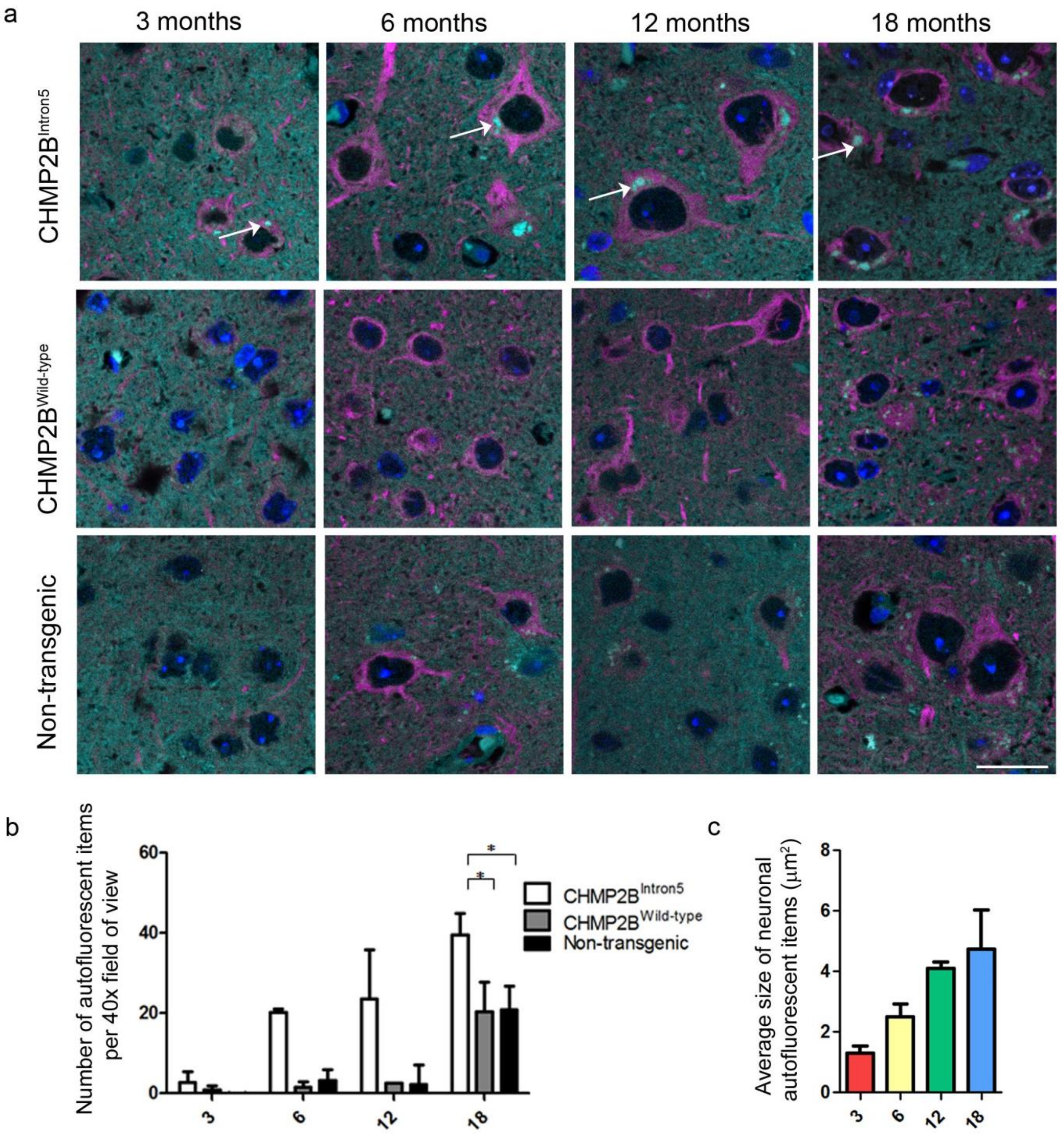

Supplementary Fig. 1 Progressive accumulation of autofluorescent aggregates in the cortex of CHMP2B^{Intron5} mice. **(a)** Spectrally unmixed images showing autofluorescent aggregates in the cortex of CHMP2B^{Intron5} mice, but not in CHMP2B$^{Wild\text{-}type}$ or non-transgenic control samples at 3, 6, 12 or 18 months of age. Nuclei are stained with DAPI (blue), neurons with β-III tubulin (magenta) and autofluorescence is shown in cyan. Arrows indicate autofluorescent aggregates. Scale bar = 20 µm. **(b)** Quantification of the number of autofluorescent items in the cortex of CHMP2B^{Intron5}, CHMP2B$^{Wild\text{-}type}$ and non-transgenic control mice. **(c)** Average size of neuronal autofluorescent items at 3, 6, 12 and 18 months in the cortex of CHMP2B^{Intron5} mice. Data are shown as mean ± SEM. Significance was determined using a two-way ANOVA, with post-hoc Bonferroni test. * = $p < 0.05$. All statistically significant differences are shown.

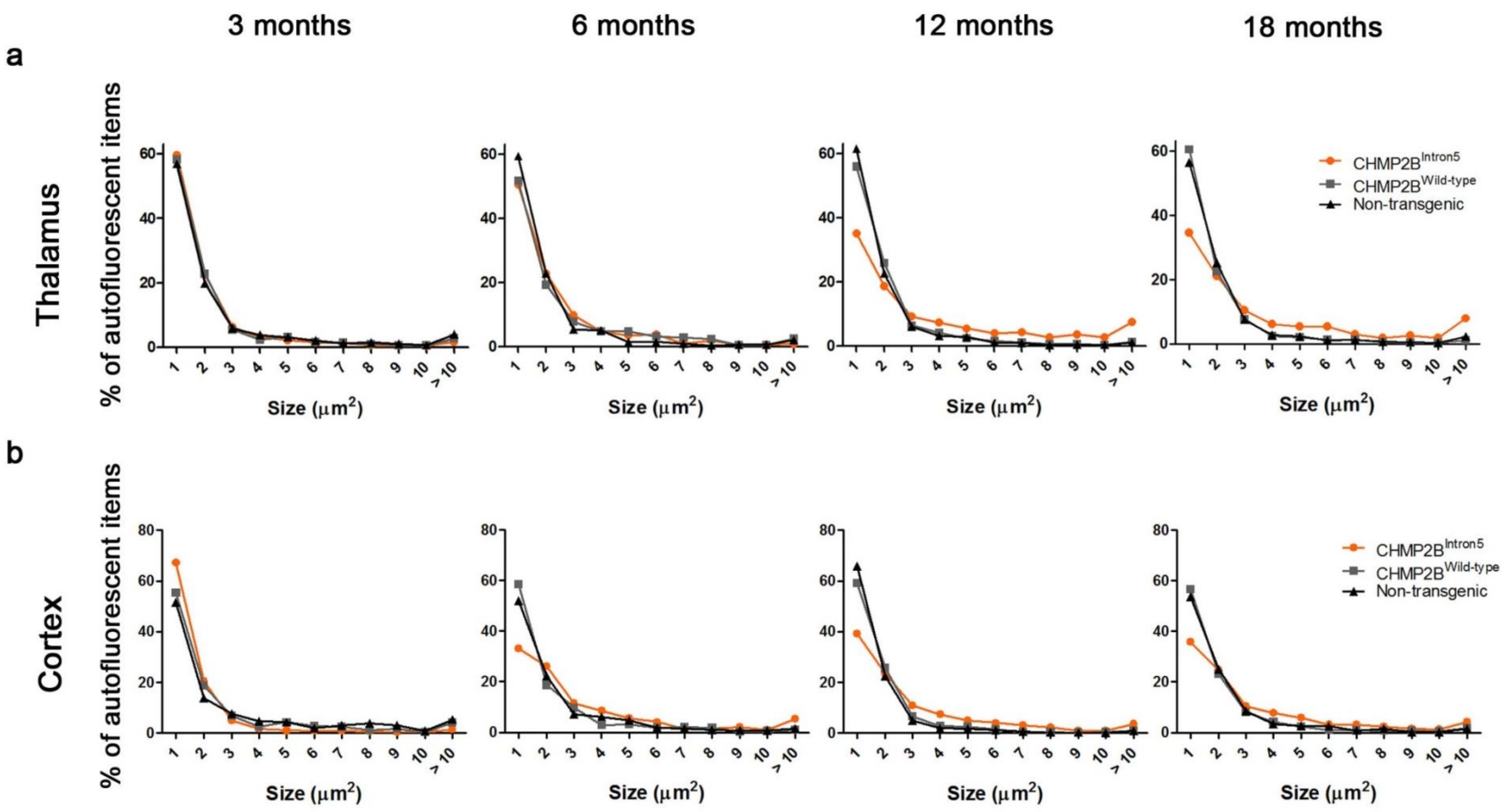

Supplementary Fig. 2 Frequency distribution of the size of all detected autofluorescent items in the **(a)** thalamus and **(b)** cortex of CHMP2B^{Intron5}, CHMP2B$^{Wild\text{-}type}$ and non-transgenic control mice at 3, 6, 12 and 18 months of age. Proportion of items > 1um^2 were analysed by one-way ANOVA with post-hoc Bonferroni's multiple comparison test showing the following significant differences: 6 month cortex CHMP2B^{Intron5} vs. CHMP2B$^{Wild\text{-}type}$ (*), 12 month cortex CHMP2B^{Intron5} vs. non-transgenic (*), 12 month thalamus CHMP2B^{Intron5} vs. non-transgenic (***), 12 month thalamus CHMP2B^{Intron5} vs. CHMP2B$^{Wild\text{-}type}$ (**), 18 month cortex CHMP2B^{Intron5} vs. non-transgenic (**), 18 month cortex CHMP2B^{Intron5} vs. CHMP2B$^{Wild\text{-}type}$ (**), 18 month thalamus CHMP2B^{Intron5} vs. non-transgenic (**), 18 month thalamus CHMP2B^{Intron5} vs. CHMP2B$^{Wild\text{-}type}$ (***).* = $p < 0.05$, ** = $p < 0.01$, *** = $p < 0.001$.

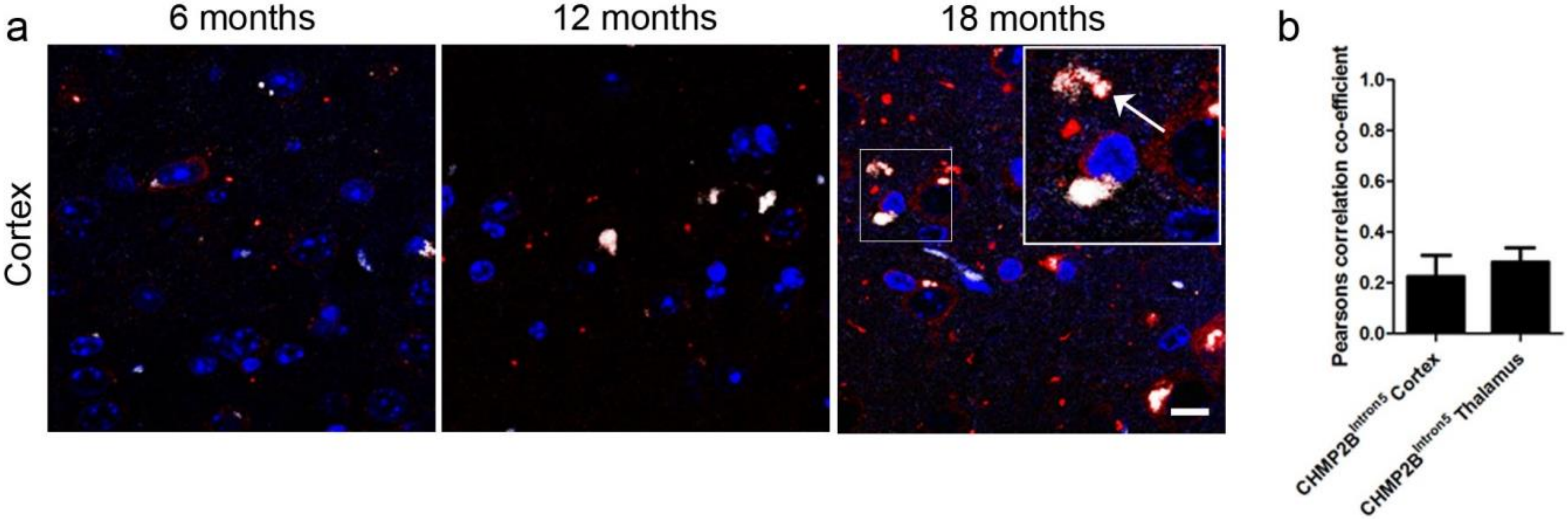

Supplementary Fig. 3 Autofluorescent aggregates and p62 positive inclusions are distinct pathologies in CHMP2B^{Intron5} mouse brain. **(a)** Spectrally unmixed images of p62 staining (red) and autofluorescence (white) in the cortex of 6, 12 and 18 month old CHMP2B^{Intron5} mice**.** p62 inclusions do not co-localise with autofluorescent items. Occasional "halos" of p62 staining can be seen surrounding the autofluorescent inclusions, see arrow in zoom box. Nuclei are stained with DAPI (blue). Scale bar = 10 µm. **(b)** Pearsons correlation of colocalisation of p62 with autofluorescent aggregates in the cortex and thalamus of 18 month CHMP2B^{Intron5} mice. Data are shown as mean ± SEM.

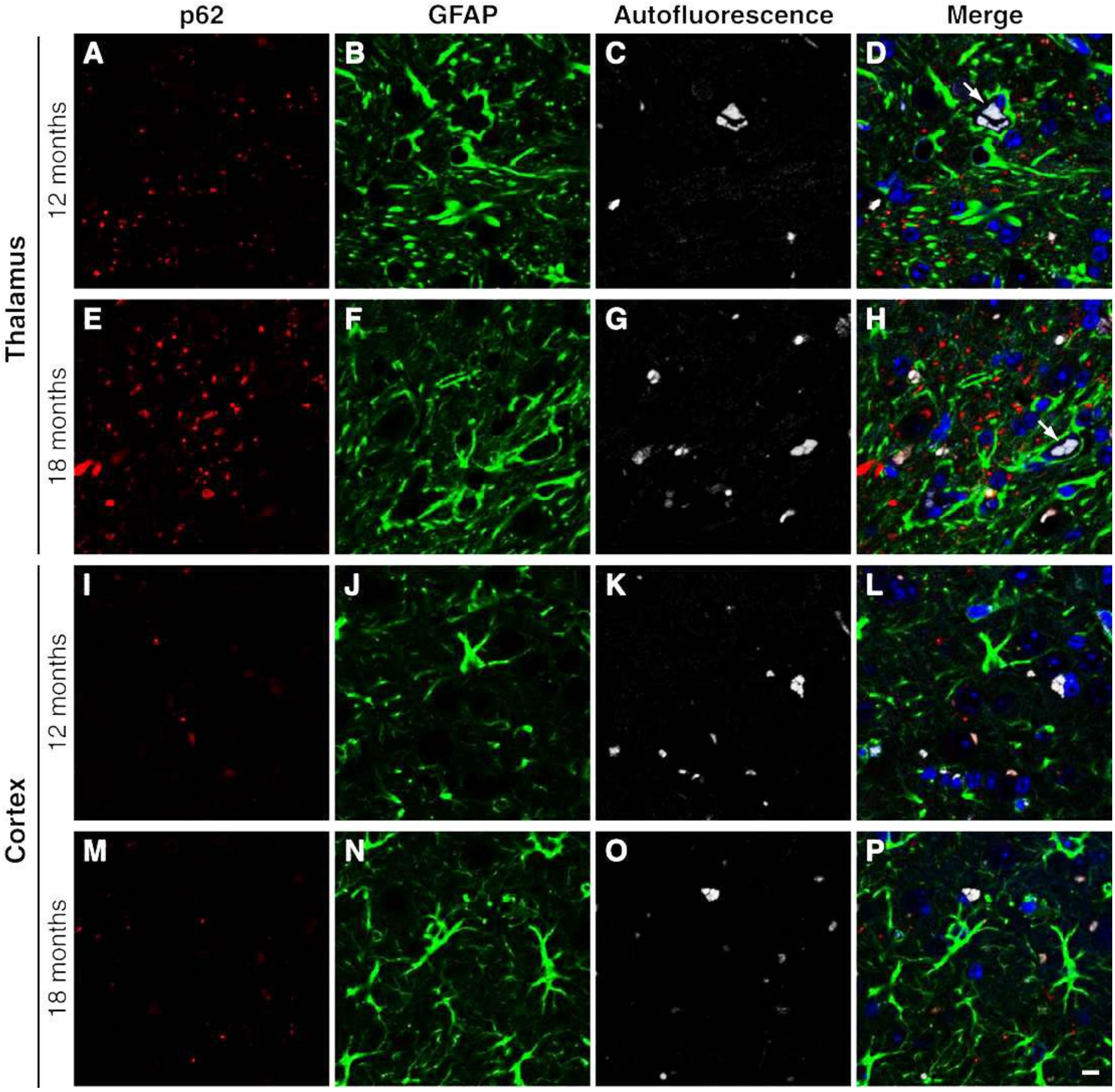

Supplementary Fig. 4 Astrocytes of CHMP2B^{Intron5} mice do not contain p62 inclusions or autofluorescent aggregates. Neither p62 inclusions (red) nor autofluorescent aggregates (white) co-localise with GFAP (green), demonstrating they are not located within astrocytes. Occasionally autofluorescent aggregates were observed that appeared surrounded by GFAP-positive processes (examples are arrowed). Nuclei are stained with DAPI (blue). Scale bar = 10 µm.

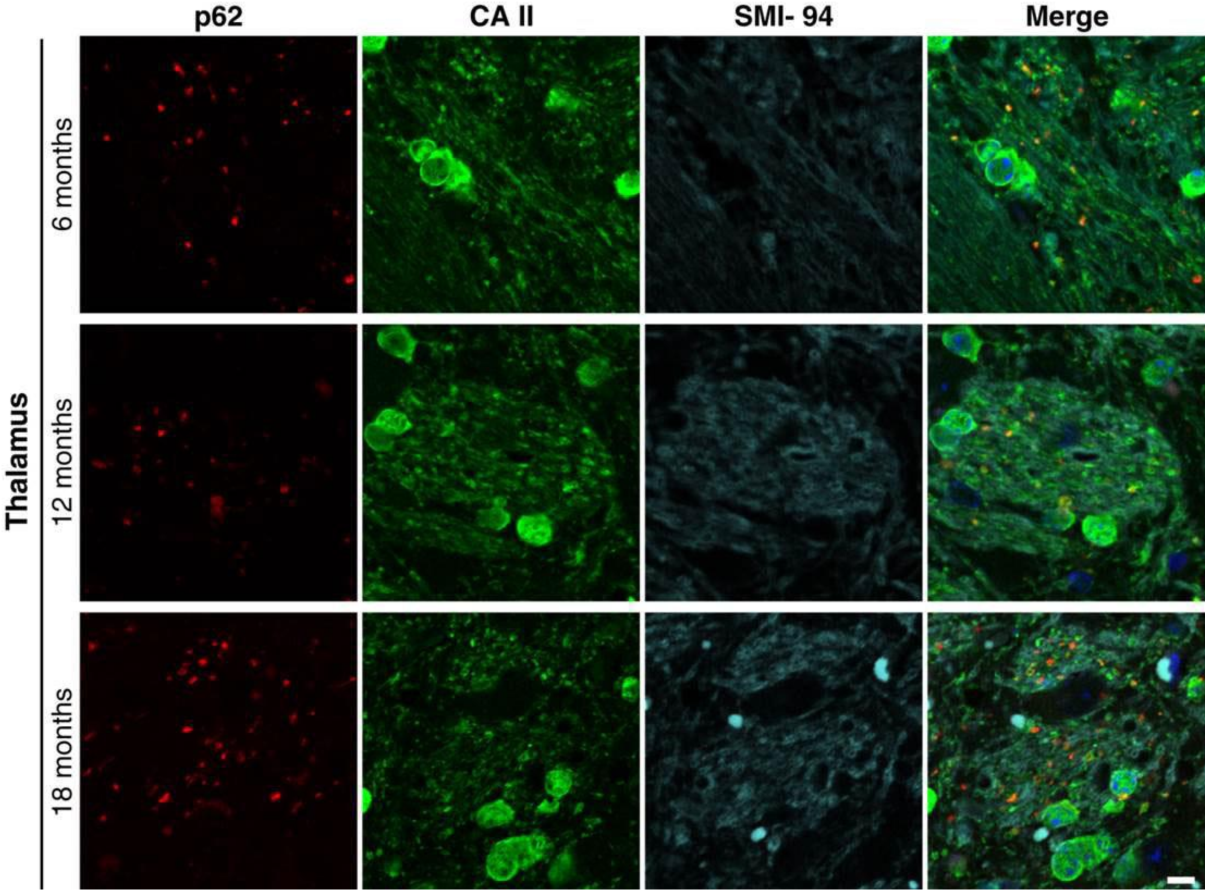

Supplementary Fig. 5 p62 inclusions co-localise with oligodendrocytic processes but not myelin in the thalamus of CHMP2B^{Intron5} mice. p62 inclusions (red) co-localise to a high degree with the oligodendrocyte marker carbonic anhydrase- II (CAII, green) in the thalamus of transgenic CHMP2B^{Intron5} mice. These inclusions are distinct from SMI-94 (cyan) indicating that they were not located within myelinated axons. Nuclei are stained with DAPI (blue). Scale bar = 10 µm.

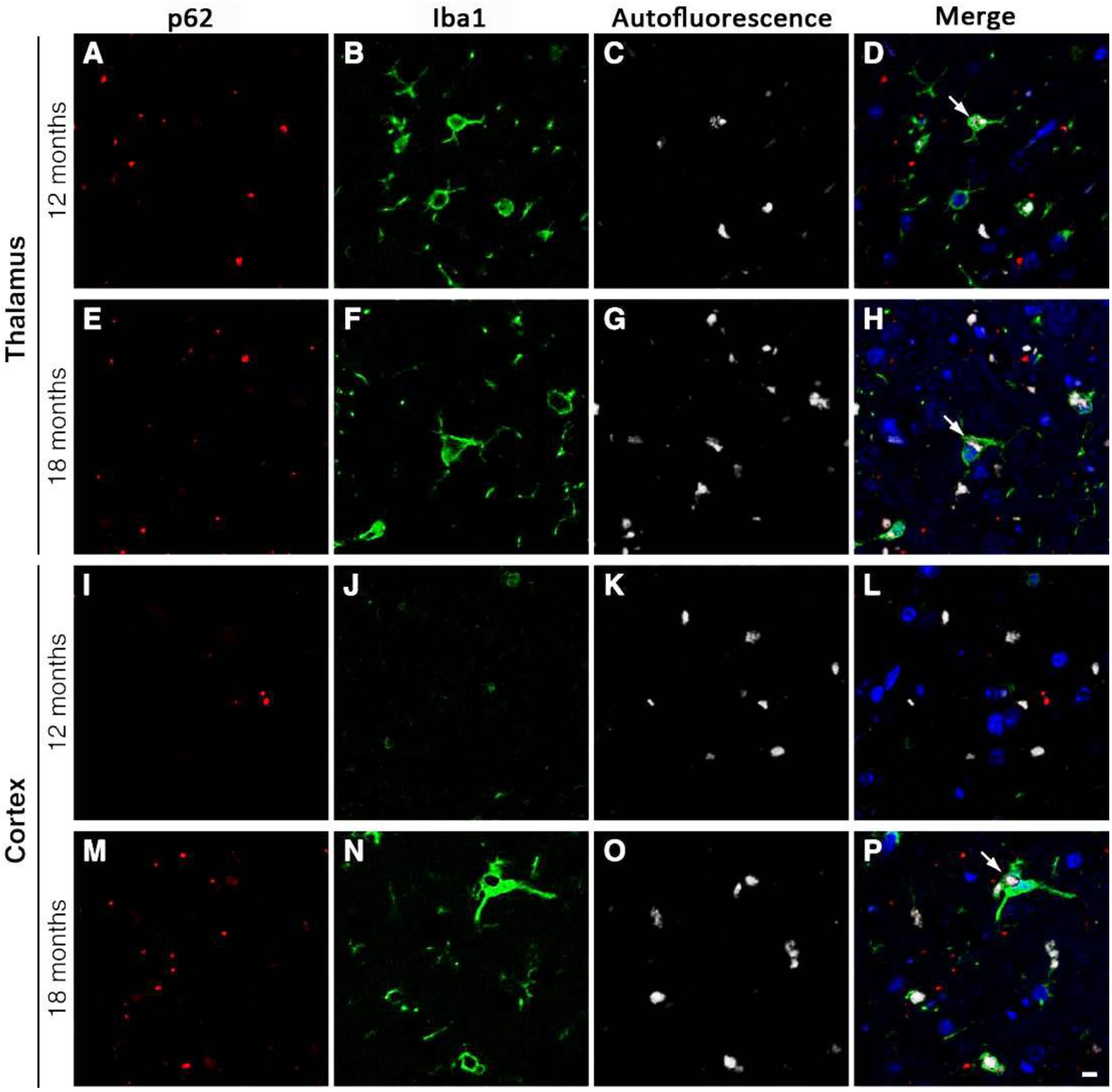

Supplementary Fig. 6 Autofluorescent aggregates, but not p62 inclusions, are found in microglia of CHMP2B^{Intron5} mice. No p62 inclusions (red) were seen in Iba1 positive microglia (green) at 12 or 18 months in either the cortex or the thalamus. Autofluorescent aggregates (white) were clearly identified within microglia (examples are arrowed). Nuclei are stained with DAPI (blue). Scale bar = 10 µm.

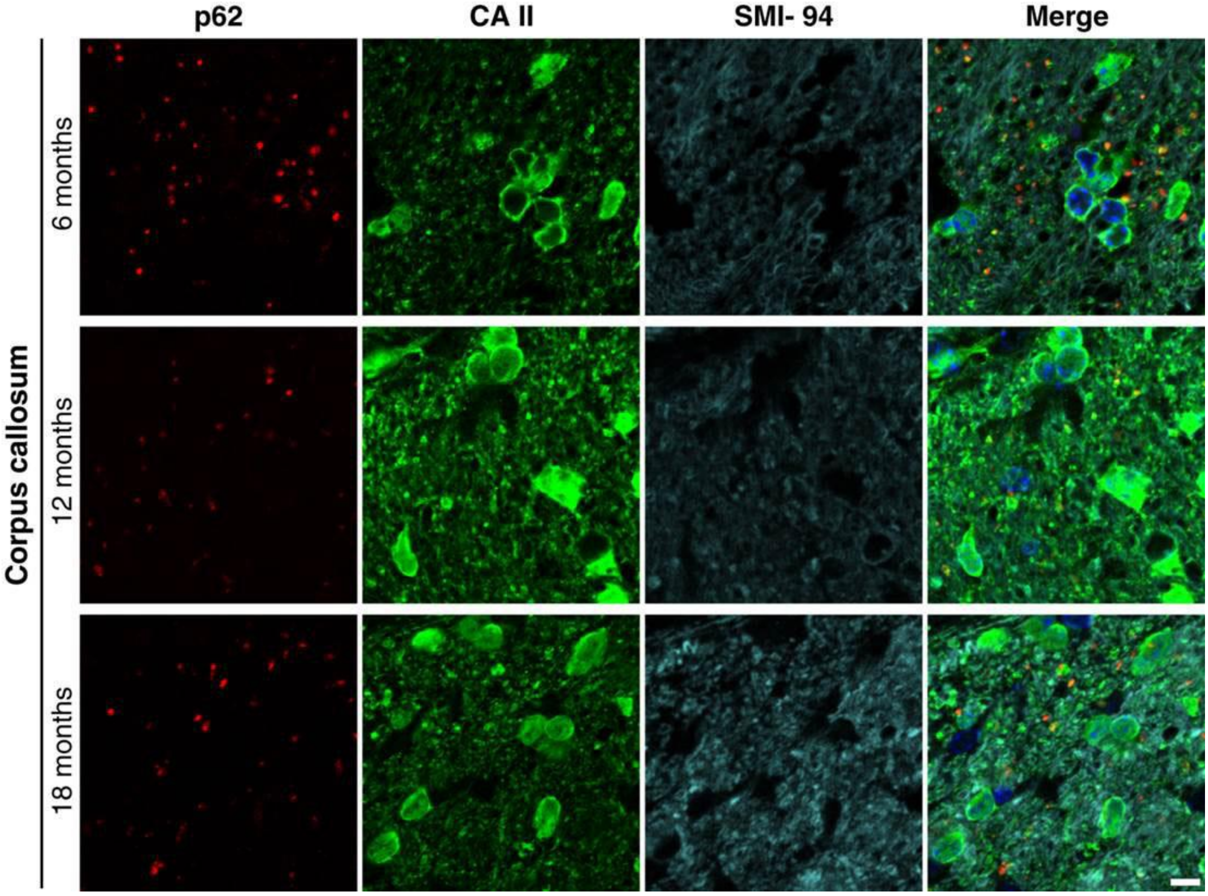

Supplementary Fig. 7 p62 inclusions co-localise with oligodendrocytic processes but not myelin in the corpus callosum of CHMP2B^{Intron5} mice. p62 inclusions (red) co-localise to a high degree with carbonic anhydrase- II (green) in the corpus callosum of transgenic CHMP2B^{Intron5} mice. These inclusions are distinct from SMI-94 (cyan) indicating that they were not located within myelinated axons but within the oligodendrocytes. Nuclei are stained with DAPI (blue). Scale bar = 10 µm.

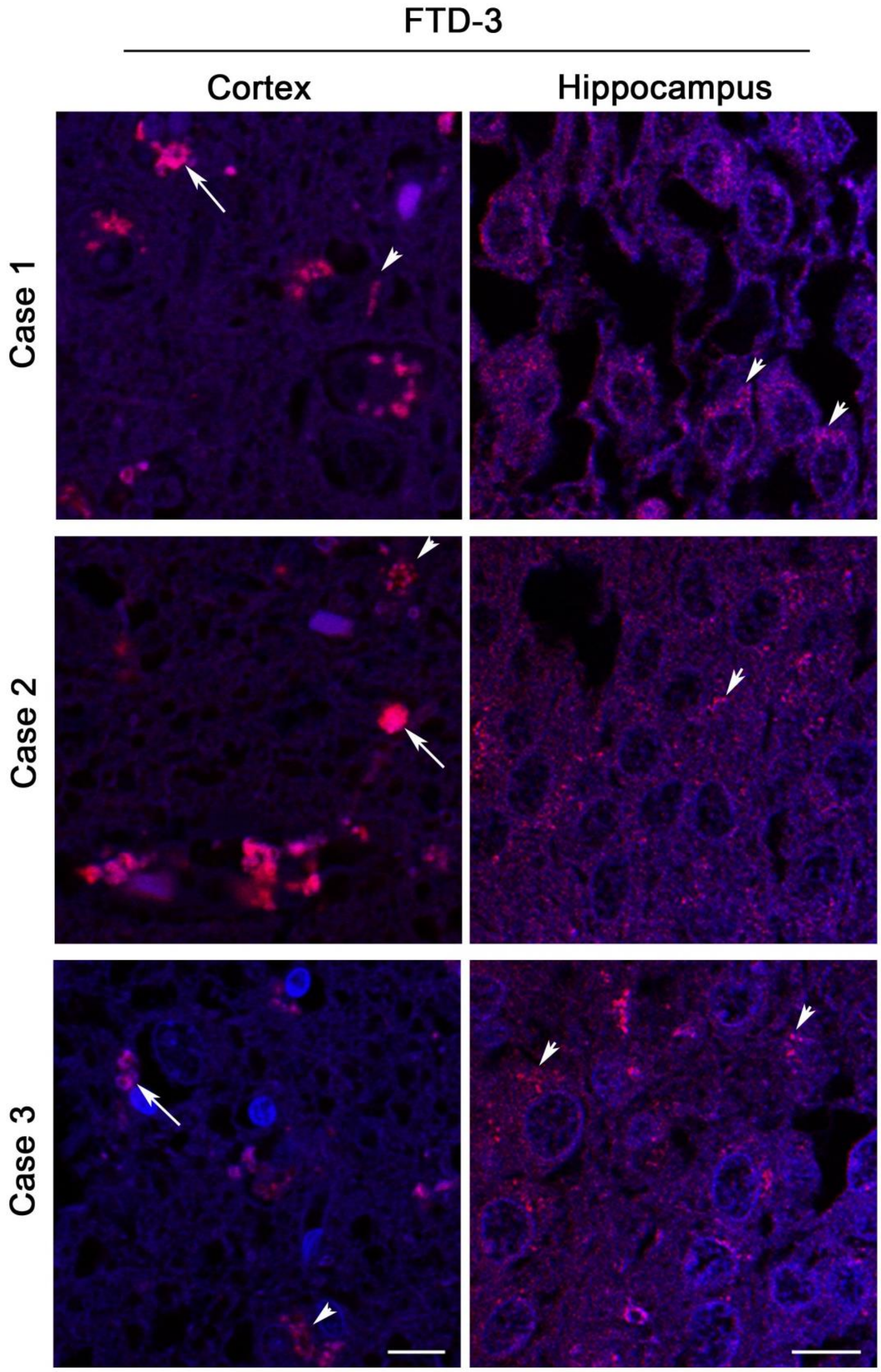

Supplementary Fig. 8 Autofluorescent aggregates are observed in the frontal cortex but not in the hippocampus of FTD-3 patient brains. Paired examples from the cortex and hippocampus of three FTD-3 patients are shown. Cases shown correspond to FTD-3 cases number 1, 2 and 3 described in table 1. Autofluorescence is shown in red, and nuclei are stained with DAPI (blue). Large autofluorescent aggregates are indicated with an arrow in the cortex of all patients. Granular lipofuscin is indicated with arrowheads in both the cortex and hippocampus for each patient example. Scale bars = 10 µm.
